# Smoking was a Possible Negative Predictor of Incident Hypertension After a Five-Year Follow-up Among a General Japanese Population

**DOI:** 10.4021/cr95w

**Published:** 2012-03-20

**Authors:** Masanori Kaneko, Eiji Oda, Hiromi Kayamori, Satomi Nagao, Hiroshi Watanabe, Takahiro Abe, Masahiro Ishizawa, Yasuyuki Uemura, Yoshifusa Aizawa

**Affiliations:** a Department of Internal Medicine, Tachikawa Medical Center, Kanda 3-2-11, Nagaoka, Niigata, Japan; b Medical Check-up Center, Tachikawa Medical Center, Nagachou 2-2-16, Nagaoka, Niigata, Japan; c Department of Cardiology, Niigata University Graduate School of Medical and Dental Sciences, Asahimachidoori 1-757, Niigata, Japan; d Department of endocrinology and metabolism, Niigata University Graduate School of Medical and Dental Sciences, Asahimachidoori 1-757, Niigata, Japan; e Department of Research and Development, Tachikawa Medical Center, Kanda 3-2-11, Nagaoka, Niigata, Japan

**Keywords:** Smoking, Hypertension, Metabolic syndrome, Insulin resistance, Inflammation

## Abstract

**Backgrounds:**

The association between cigarette smoking and hypertension is controversial. The aim of this study is to investigate the association between smoking and incident hypertension.

**Methods:**

This is a post-hoc five-year follow-up study in a general Japanese population. Logistic regressions were performed using incident hypertension as an outcome and smoking status as an independent predictor adjusting for sex, age, body mass index (BMI), total cholesterol, high-density lipoprotein (HDL) cholesterol, triglycerides, fasting plasma glucose (FPG), drinking status, and diabetes in 1,297 subjects without hypertension at baseline.

**Results:**

The incidence of hypertension was 16.9% vs. 27.6% (smokers vs. nonsmokers, P = 0.01) in men and 0.0% vs. 16.9% (smokers vs. nonsmokers, P = 0.03) in women. The odds ratio (OR) (95% confidence interval (CI)) of incident hypertension was 0.38 (0.19 - 0.76) (P = 0.006) for smokers at baseline, 0.33 (0.16 - 0.68) (P = 0.003) for continuing smokers, and 2.11 (0.33 - 13.45) (P = 0.4) for ex-smokers. Age (OR = 1.52, P < 0.0001), BMI (OR = 1.46, P < 0.0001), and FPG (OR = 1.23, P = 0.007) were other independent predictors of incident hypertension.

**Conclusions:**

Smoking was a possible significant negative predictor of incident hypertension in a general Japanese population.

## Introduction

Cigarette smoking is an indisputable and devastating toll on public health and one of the major risk factors of cardiovascular disease (CVD) [1-5]. Hypertension is the most important risk factor of CVD among five components of metabolic syndrome (MetS) [6-8] in Japanese [4, 9]. The population-attributable fraction of CVD mortality due to smoking or hypertension was 35.1% for men and 22.1% for women in Japanese [4]. Oh et al reported that cigarette smoking was independently associated with MetS among Korean men [10]. Smokers have abnormalities in lipoprotein metabolism and there is some evidence that they are at greater risk than non-smokers of becoming insulin resistant and hyperinsulinemic [11-13]. Smoking is associated with increases in various markers of systemic inflammation including high-sensitivity C-reactive protein and white blood cell count, and cessation of smoking is associated with a decreased inflammatory response, which may represent one mechanism responsible for the reduced CVD risk in these subjects [14, 15]. As for the relationship between smoking and hypertension, results from epidemiologic studies have generally shown smokers to have lower blood pressure than non-smokers [16] although cigarette smoking produces an acute rise in blood pressure. Some studies in Asians reported that the incidence of hypertension was higher in never smokers or past smokers than in current smokers and cessation of smoking might result in hypertension [17, 18]. On the contrary, other studies among Westerners suggest that cigarette smoking may be a positive risk factor for the development of hypertension [19, 20].

## Subjects and Methods

### Subjects

This community-based, post-hoc study was based on annual health examinations organized by the Niigata Health Foundation (Niigata, Japan) [21]. Informed consent was obtained from each participant. The subjects in the present study were 1,297 men and women whose information about smoking status was available and who were without hypertension at baseline among subjects who took a baseline examination in 1998 and repeated the examination in 2003. The present study was approved by the ethics committees at Tachikawa Medical Center and at Niigata University Graduate School of Medical and Dental Sciences.

### Measurements

After an overnight fast, blood samples were obtained to measure total cholesterol, triglycerides, high-density lipoprotein (HDL) cholesterol, and fasting plasma glucose (FPG). Blood pressure was measured in supine position after a five minutes rest. Body mass index (BMI) was calculated as weight in kilograms divided by the square of height in meters. Hypertension was defined as SBP ≥ 140 mmHg and/or DBP ≥ 90 mmHg or antihypertensive medication.

### Statistical analysis

Logistic regressions were performed using incident hypertension as a dependent variable and sex, age, BMI, total cholesterol, HDL cholesterol, triglycerides, FPG, diabetes, drinking status, and smoking status at baseline as independent variables. Numerical variables were divided by 1 SDs for standardization. Similar logistic regressions were repeated for continuing smoking and ex-smoking at follow-up instead of smoking status at baseline. Statistical analyses were performed with Dr SPSS-2 (SPSS Japan Inc., Tokyo, Japan). Means were compared with t-tests and prevalence or incidence was compared with chi-squared tests. P values of less than 0.05 were considered as significant.

## Results

Basal data are presented in Table 1 and 2. The subjects were 228 men aged 41 - 92 years and 1,069 women aged 40 - 82 years. Incident hypertension developed in 17.1% of subjects through five years. The incidence of hypertension was 12.4% in smokers vs. 26.0% in nonsmokers in men (P = 0.01) and 0.0% in smokers vs. 16.9% in nonsmokers in women (P = 0.03). Among 128 smokers at baseline, 123 subjects continued smoking through follow-up and five subjects had quitted smoking by follow-up.

**Table 1:** Baseline Data and Incident Hypertension by Smoking Status in Men

	smoker	nonsmoker	P
n	105	123	
incident hypertension (%)	12.4	26.0	0.01
age (years)	61.6 ± 10.5	64.8 ± 8.3	0.01
body mass index (kg/m^2^)	22.3 ± 2.6	22.1 ± 2.4	0.6
systolic blood pressure (mmHg)	122.4 ± 10.2	124.1 ± 10.4	0.2
diastolic blood pressure (mmHg)	74.7 ± 7.5	75.7 ± 7.4	0.3
total cholesterol (mg/dL)	203.1 ± 39.0	204.2 ± 32.4	0.8
HDL cholesterol (mg/dL)	55.9 ± 13.6	63.0 ± 17.4	0.0008
triglycerides (mg/dL)	124.2 ± 105.8	100.0 ± 70.1	0.04
fasting plasma glucose (mg/dL)	88.8 ± 8.8	92.9 ± 11.2	0.003
everyday drinker (%)	60.0	52.8	0.1
diabetes (%)	2.3	6.1	0.3

mean ± SD or %.

**Table 2:** Baseline Data and Incident Hypertension by Smoking Status in Women

	smoker	nonsmoker	P
n	23	1,046	
incident hypertension (%)	0.0	16.9	0.03
age (years)	55.4 ± 8.8	59.4 ± 8.6	0.03
body mass index (kg/m^2^)	21.9 ± 2.8	22.3 ± 2.7	0.5
systolic blood pressure (mmHg)	118.1 ± 11.3	121.2 ± 11.0	0.2
diastolic blood pressure (mmHg)	71.8 ± 8.2	73.6 ± 7.8	0.3
total cholesterol (mg/dL)	212.6 ± 34.9	221.8 ± 33.1	0.2
HDL cholesterol (mg/dL)	67.7 ± 28.1	66.1 ± 14.8	0.6
triglycerides (mg/dL)	106.1 ± 55.0	93.8 ± 46.5	0.2
fasting plasma glucose (mg/dL)	86.8 ± 17.2	88.0 ± 9.4	< 0.0001
everyday drinker (%)	17.4	7	0.06
diabetes (%)	4.3	1.1	0.1

mean ± SD or %.

The odds ratios (ORs) (95% confidence interval (CI)) of incident hypertension are presented in Table 3-5. The OR (95% CI) of incident hypertension was 0.38 (0.19 - 0.76) (P = 0.006) for smoker at baseline, 0.33 (0.16 - 0.68) (P = 0.003) for continuing smoker, and 2.11 (0.33 - 13.45) (P = 0.4) for ex-smoker. Age, BMI, and FPG were significantly positively associated with incident hypertension. However, HDL cholesterol was not significantly associated with incident hypertension.

**Table 3:** Odds Ratios* of Incident Hypertension Using Smoker at Baseline as an Independent Variable

	odds ratio*	95% confidence interval	P
smoker at baseline	0.38	0.19 - 0.76	0.006
female sex	0.91	0.55 - 1.51	0.7
age	1.52	1.29 - 1.79	< 0.0001
body mass index	1.46	1.25 - 1.71	< 0.0001
total cholesterol	0.92	0.77 - 1.09	0.3
HDL cholesterol	1.02	0.85 - 1.23	0.8
triglycerides	1.16	0.99 - 1.35	0.06
fasting plasma glucose	1.23	1.06 - 1.42	0.007
everyday drinker	1.48	0.93 - 2.37	0.1
diabetes	0.40	0.10 - 1.57	0.2

* Odds ratios for 1 SD increment for numerical variables and those for the positive status compared with the negative status for categorical variables.

**Table 4:** Odds Ratios* of Incident Hypertension Using Continuing Smoker as an Independent Variable

	odds ratio*	95% confidence interval	P
continuing smoker	0.33	0.16 - 0.68	0.003
female sex	0.89	0.54 - 1.48	0.7
age	1.51	1.28 - 1.79	< 0.0001
body mass index	1.46	1.25 - 1.71	< 0.0001
total cholesterol	0.92	0.77 - 1.09	0.3
HDL cholesterol	1.02	0.84 - 1.23	0.8
triglycerides	1.17	1.00 - 1.36	0.05
fasting plasma glucose	1.22	1.05 - 1.41	0.01
everyday drinker	1.48	0.93 - 2.37	0.1
diabetes	0.41	0.10 - 1.61	0.2

* The same as Table 3.

**Table 5:** Odds Ratios* of Incident Hypertension Using Ex-Smoker as an Independent Variable

	odds ratio*	95% confidence interval	P
ex-smoker	2.11	0.33 - 13.45	0.4
female sex	1.28	0.80 - 2.05	0.3
age	1.54	1.30 - 1.81	< 0.0001
body mass index	1.48	1.26 - 1.73	< 0.0001
total cholesterol	0.91	0.77 - 1.08	0.3
HDL cholesterol	1.04	0.86 - 1.26	0.7
triglycerides	1.15	0.98 - 1.34	0.08
fasting plasma glucose	1.24	1.07 - 1.43	0.005
everyday drinker	1.41	0.88 - 2.24	0.2
diabetes	0.42	0.11 - 1.63	0.2

* The same as Table 3.

## Discussion

Oh et al reported that cigarette smoking was independently associated with MetS [6-8] among Korean men and that triglycerides, HDL cholesterol, and waist circumference were the main contributors to the association between smoking and MetS [10]. They could not find an association of smoking with FPG or blood pressure in their cross-sectional study [10]. Facchini et al reported that cigarette smoking is associated with increases in triglycerides levels and decreases in HDL cholesterol levels and that these changes are secondary to insulin resistance or hyperinsulinemia [11]. Farin et al reported that dyslipidemia (high triglycerides and low HDL cholesterol) previously attributed to smoking occurs primarily in those smokers who are also insulin resistant [12]. Among five components of MetS, the relationship between HDL cholesterol and hypertension is unique and controversial [22, 23]. HDL cholesterol and blood pressure do not significantly correlate with each other [23] Halperin et al reported that HDL cholesterol was negatively associated with incident hypertension [22]. However, HDL cholesterol was positively associated with hypertension by multivariate logistic regression in our previous cross-sectional study [23].

In the present study, smoking was a significant negative predictor of incident hypertension adjusting for sex, age, BMI, total cholesterol, HDL cholesterol, triglycerides, FPG, drinking status, and diabetes in 1,297 general Japanese subjects. However, smoking was not a significant predictor of incident hypertension further adjusted for blood pressure per se. Age, BMI, and FPG were other independent predictors of incident hypertension. But, HDL cholesterol was not significantly associated with incident hypertension.

Green et al reported that blood pressure was higher among never smokers and past smokers than among current smokers and that the difference could not be explained by various potentially confounding factors, such as BMI, ethnic origin, alcohol and coffee intake, and participation in leisure time sports [16]. Cotinine, which is a metabolite of nicotine and reflects a daily intake of nicotine, may be one of possible links between cigarette smoking and the lower blood pressure because Benowitz et al reported the inverse relationship between serum cotinine concentration and blood pressure in smokers [24]. Mental relaxation brought by cigarette smoking may be another possible mechanism of blood pressure lowering. Lee et al investigated the effects of smoking cessation on changes in blood pressure and incidence of hypertension in 8,170 healthy Korean male employees at a steel manufacturing company and reported that the adjusted relative risks (RR) (95% CI) of hypertension in those who had quit smoking for 1, 1 to 3, and ≥ 3 years were 0.6 (0.2 - 1.9), 1.5 (0.8 - 2.8), and 3.5 (1.7 - 7.4), respectively, compared with current smokers [17]. The trends for increased risk of hypertension for longer periods of smoking cessation were observed in subgroups of those who maintained weight as well as those who gained weight after smoking cessation [17]. Bowman et al conducted a prospective cohort study among 28,236 women in the Women's Health Study who were initially free of hypertension, CVD, and cancer in the United States [19]. During a median of 9.8 years, there were 8,571 (30.4%) cases of incident hypertension. The age-adjusted hazard ratios (HR) (95% CI) of developing hypertension among never, past, and current smokers of 1 to 14, and > or =15 cigarettes per day were 1.00 (reference), 1.04 (0.99 - 1.09), 1.00 (0.90 - 1.10), and 1.10 (1.01 - 1.19), respectively. In multivariable models further adjusting for lifestyle, clinical, and dietary variables, the corresponding HR (95% CI) were 1.00 (reference), 1.03 (0.98 - 1.08), 1.02 (0.92 - 1.13), and 1.11 (1.03 - 1.21), respectively [19]. Halperin et al analyzed 13,529 male participants from the Physicians' Health Study free of baseline hypertension and CVD in the United States [20]. Over a median follow-up of 14.5 years, 4,904 (36.2%) men developed hypertension. Compared with never smokers, past smokers and current smokers had corresponding multivariate-adjusted RR (95% CI) of 1.08 (1.01 - 1.15) and 1.15 (1.03 - 1.27) of developing hypertension. The risk for smokers did not appear to differ based on number of cigarettes smoked daily [20]. These prospective cohort data in the United States suggest that cigarette smoking may be a modest risk factor for the development of hypertension. In a prospective study over a mean 7.4 years among middle-aged and elderly Turkish adults (2,427 men and women, aged 45.8 ± 11.7 years), Onat et al reported that relative risk for incident hypertension of current vs. never smoking was marginally reduced in women (RR (95% CI) of 0.74 (0.52 - 1.05), P = 0.058) and both genders combined (RR (95% CI) of 0.80 (0.64 - 1.007), P = 0.054) [18]. The risk of incident hypertension for past smokers was marginally higher (RR (95% CI) of 1.33 (0.98 - 1.81), P = 0.054) compared with never smokers and significantly higher (RR (95% CI) of 1.66 (1.24 - 2.23), P < 0.001) compared with current smokers [18]. Dochi et al reported that OR (95% CI) of incident hypertension for smoking was 1.13 (1.03 - 1.23) in Japanese male workers at a steel company [25]. Tamura et al reported that ex-smokers had increased levels of body weight, systolic and diastolic blood pressure, total cholesterol, triglyceride, FPG, and HDL cholesterol compared to continuing smokers [26]. Thus, the relationship between cigarette smoking or cessation of smoking and incident hypertension is controversial.

In the present study, smoking was suggested to be negatively associated with incident hypertension in a general Japanese population. Therefore, the coincidence of smoking and hypertension suggests some genetic predisposition to hypertension and CVD risk. Although the number of ex-smokers was too small to obtain statistically significant results, cessation of smoking may possibly increase the risk of hypertension. Therefore, though cessation of smoking should be recommended to every smoker, the development of hypertension should be strictly watched after cessation of smoking.

### Limitations

This is a post-hoc study with a relatively short follow-up period and the subjects were not randomly selected from the community. The number of subjects, especially those who quitted smoking, was small and quantitative information on smoking status was not included in the present study. Thus, the conclusions of the present study are hypothetical and more studies including detailed temporal and quantitative information about smoking status among a general population are required to confirm the present results.
